# Narrative review of women’s health in Iran: challenges and successes

**DOI:** 10.1186/s12939-016-0316-x

**Published:** 2016-02-16

**Authors:** Hassan Joulaei, Najmeh Maharlouei, Kamran Bagheri lankarani, Alireza Razzaghi, Maryam Akbari

**Affiliations:** Shiraz HIV/AIDS Research Center, Shiraz University of Medical Sciences, Shiraz, Iran; Health Policy Research Center, School of Medicine, Shiraz University of Medical Sciences, Shiraz, IR Iran; Guilan Road Trauma Research Center, Guilan University of Medical Science, Guilan, Iran

## Abstract

**Background:**

In each society, the health of women in different periods may be endangered by an unequal distribution of resources, facilities, and gender prejudices. The present study evaluated the time trend of Iranian women’s health between 1990 and 2013.

**Methods:**

This narrative review includes an integration and descriptive summary of the existing evidence on trends and criteria of different aspects of women’s health from social determinant point of view. The evidence was drawn from peer-reviewed, cross-national or large-scale studies, official sources of the Ministry of Health, reviews, and online scientific databases published between 1990 and 2013.

**Results:**

The average life expectancy of Iranian women has increased from 44.15 years in 1960 to 75.75 years in 2012; in most deprived provinces of Iran, however, this criterion is about 67.3 years, and in the capital it is 75.8 years. In 2011, 43.37 % of DALYS, 36.21 % of YLL, and 1.92 % of YLD were dedicated to women; these figures were 3.63 % lower than they were in 2003. Although a significant reduction has occurred in maternal mortality rate, which dropped from 83 to 23 per 100,000 between 1990 and 2013, there is no equal distribution in maternal mortality across the country as manifested by the unfavorable conditions of border provinces (SD = 19.2). The prevalence of HIV/AIDS is an alarming health problem among Iranian females, increasing approximately 546 % between 2007 and 2015. As for mental health, depression in women was ranked first among diseases in 2011 compared to a second place ranking in 2003. As regards social health, the delinquency of women has increased in recent years compared to men with women committing more crimes related to drugs and actions against virtue. The annual report of the United Nations for the gender gap index in 2013 ranked Iran as 130 among 136 countries (from 0.622 in 2000 to 0.584 in 2013).

**Conclusion:**

Generally, over the last three decades, the health indices of Iranian women have grown in aspects of physical, mental, and social health. Remarkable differences can be seen among female health indices based on geographic location and in comparison with men. To promote an improved health status for Iranian women, the root causes of the discrepancies must be identified and a comprehensive national plan must be established.

## Background

Globally, health is a precondition of the fulfillment of sustained development for which healthy humans play a key role. In this context, the health of women is of special importance, because they comprise half the world’s population and play a pivotal role in family health and the development of societies. In other words, researchers believe that it is impossible to achieve the universal promotion of health and hygiene without women’s health [1]. Health and its equitable distribution among various sectors of society have been principal themes of the World Health Organization, many other international agencies, and researchers over the past two decades. For this reason, women’s health and its effective role in achieving the millennium goals of reduced mortality rates among mothers, gender equality, and the empowerment of women have drawn the special attention of the United Nations and its member states [2]. Women’s health has been regarded as topmost among the activities and goals of health systems in communities and a benchmark for the development of countries. Nonetheless, according to the reports published by the United Nations Organization, women’s health is vulnerable and deserves the special attention of policymakers [3].

According to World Health Organization reports, women are vulnerable for various reasons, such as their roles in family and society and various physiologic conditions including puberty, menstruation, pregnancy, and menopause. The female community is considered a high risk population because of the inequitable distribution of resources and opportunities, high poverty, hunger, and malnutrition rates, and gender discrimination against women [4]. The well-being of a community is assessed and monitored on the basis of its health status standard. In this context, the criteria representing the perspective of the female community and reflecting the existing health gaps in women’s health include burden of disease, maternal mortality rates [5], repeated family violence [6], unequal job opportunities [7], poverty and disproportionate income [8], inaccessibility of educational facilities [9], intensive stress [10], and depression and isolation [7].

In Iran, like in many other countries, traditional differences exist between men and women in how they benefit from facilities [11]. In recent years, the Islamic Republic of Iran, like other states wherein rapid changes in social, economic and population dynamics have taken place, has undergone transformations which are considered as criteria for the successful assessment of the human development index. This is indicated by a growth rate of 0.443 in 1980 compared with 0.742 in 2012, which is equal to 1.05 % or a global ranking of 76 [12]. Although this is an acceptable pace, its impact on reducing gender inequality remains unknown. The present study attempts to answer this question so as to provide concrete evidence to those involved in policy and decision making to aid the establishment of justice and reduce health-related inequalities. In addition, it explores the trends and criteria of different aspects of women’s heath in the period 1990-2013.

## Materials and methods

This study is a narrative review that includes an integration and descriptive summary of the existing evidence on trends and criteria of different aspects of women’s health.

**Fig. 1:**
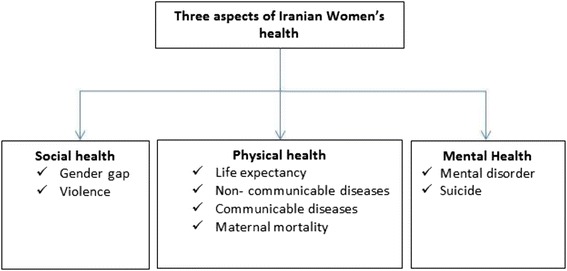
Algorithm of the searching strategy

A search for literature from 1990 to 2013 was conducted in medical databases including PubMed, Medline, and Google Scholar and Iranian Farsi sources including Iran medex, SID, and MagIran. Based on the World Health Organization’s definition, three aspects of health were considered: “physical health”, “mental health”, and “social health.” The algorithm of the searching strategy for those aspects is. (Fig. 1)

Considering the above-mentioned aspects, and with focus on the outcome of each aspect, keywords used in the searches were “Iranian women” or “Iranian female” and “inequality”, “life expectancy”, “burden of diseases”, “diabetes”, “hypertension”, “BMI”, “physical activity”, “maternal mortality”, “HIV/AIDS”, “STIs”, “communicable diseases”, “mental disorders”, “suicide”, “gender gap”, or “violence.” To further access some topics, other data sources such as the Statistical Center of Iran which is considered the country’s most reliable statistical center, population and housing censuses, and the official reports of the Ministry of Health and Medical Education, the World Health Organization, and the United Nations Development Program were also accessed. The time period for the search was limited to 2000 and later. In this study, articles or documents published after 2000 were considered. Regarding the WHO definition; in this study equity is defined as the absence of any differences among groups of people that would be avoidable or treatable.

Because of the myriad health–related terms and extensive information about women’s health in Iran, this report was confined to a limited number of terms related to ill health as the outcome of social and health systems so that women’s health in Iran could be more easily depicted.

## Results

The results of the current study are presented in three parts, physical health, mental health, and social health indicators, as follows.

**Fig. 2:**
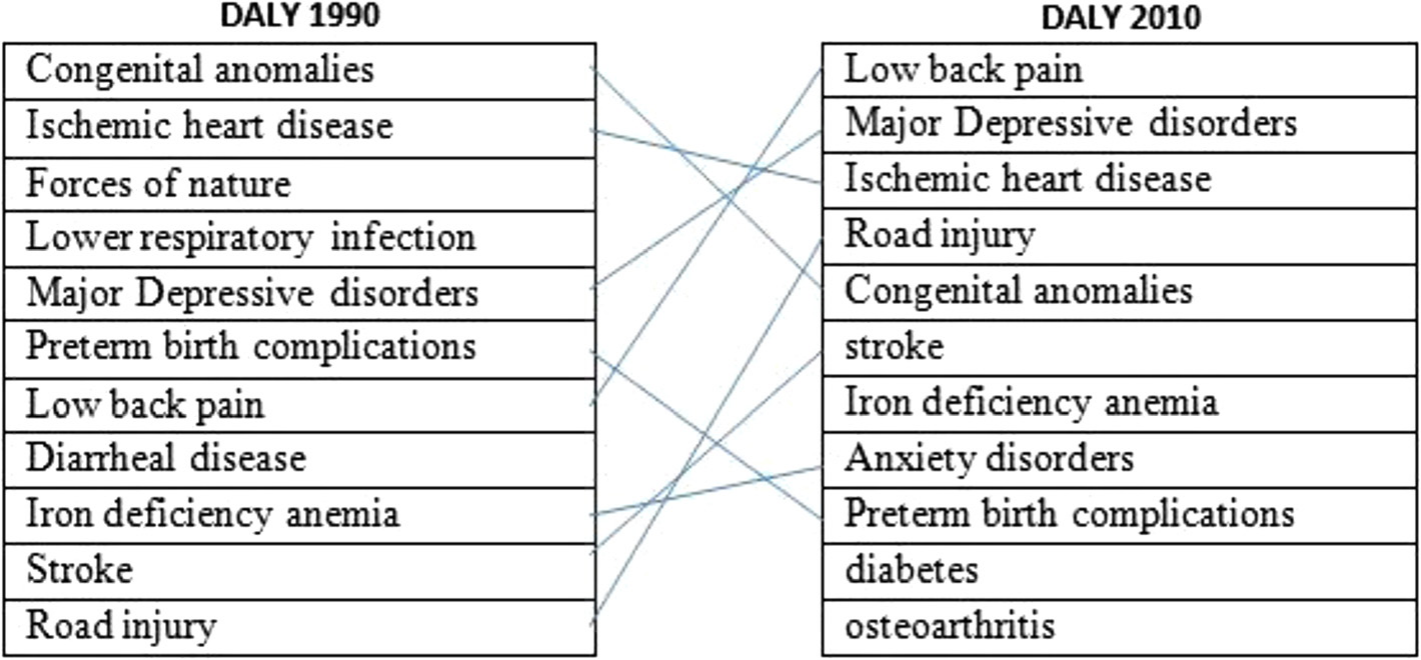
Cause of DALYs for female, all age, Iran, 1990–2010
  Studies of elderly Iranian people showed that 17 % of elderly men and 25 % of elderly women suffer from heart disease and 42 % of aged men and 46 % of aged women have high blood pressure. The prevalence of hyperglycemia was reported as 63.6 % of aged women and 42.6 % of elderly men [19]. Study results revealed that the rate of osteoporosis in females is more than triple that of males (56.3 % vs. 16.7 %, respectively). The prevalence of pain involving weight-bearing and non-weight-bearing joints among Iranian women aged over 50 years is 6 times higher than their male counterparts. Smokers comprise 39 % of the aged population, of which 38.9 % are men and 29.1 % are women [20]. Furthermore, reports indicated that physical problems (43 % vs. 39 %) and nostalgic and psychological problems (8.17 % vs. 3.25 %) are higher among aged women than elderly men [21].
  **Communicable diseases:** HIV/AIDS has a growing trend in Iran. The number of people living with HIV (PLWH) is estimated to increase from 43,000 in 2006 to 110,000 in 2016. According to the 2014 UNAIDS report, the number of male PLWH will increase by 60.4 % by 2016, while this rate for female PLWH is estimated to surge by 546 %. The third wave of transmission has provoked an increasing trend of HIV among Iranian females; the rate of HIV is 4.5 % for female sex workers [22]. Based on a study done in 2014, the prevalence of sexually transmitted infections [23] associated with symptoms in women (39.9 %) is more than double that of men (17.6 %) [24]. Furthermore, genital ulcers in Iranian females are estimated to be 4 % while in males it is less than 0.4 % [25]. Nasirian et al. showed that 29.7 % of men had experienced at least one STIs-associated symptom during the year prior to the study, while this rate was 81.8 % for women [24]. Giardiasis is the most prevalent parasitic infection in Iran, and studies showed a higher prevalence in males (15.1 %) than in females (13.9 %). Listeriosis is another infectious disease common among Iranian females, seen in 36 % of women with a history of spontaneous abortion and 18 % of those with normal full-term deliveries [26].
  **Maternal health:** Maternal mortality per 100,000 live births in Iran has shown a diminishing trend over the past decade (Fig. 3).This is evidenced by a reduction in the maternal mortality rate from 83 to 23 per 100,000 live births between 1990 and 2013, which varied between different provinces in Iran [27, 28]. Undoubtedly, there was no even distribution in maternal mortality rates across the country as manifested by the unfavorable conditions found in border provinces (SD = 19.2) [29, 30]. Studies have shown that 90 % of the maternal mortality rate is related to defective emergency midwifery services. Of these, 73 % was due to poor service quality and 25 % was associated with unavailable facilities; 27 % mortality due to hemorrhage has remained of topmost concern over the past two decades. All criteria associated with mothers’ health have been improved over the past 20 years except for the rate of cesarean delivery [31].

1. Physical health indicators
  Life expectancy: Life Expectancy (LE) is an important determinant associated with health-related results and is a so-called outcome indicator that plays an important role in public health [13]. Iranian women have higher LE than men (77.8 years (75.3 - 80.2) vs. 71.6 years (68.5 - 74.6), respectively) [14]. The LE of women increased from 44.15 years in 1960 to 75.75 years in 2012. However, an important issue is the inter-provincial differences. While the LE of women in the most deprived province of Iran (Sistan and Baluchestan) is about 67.3 years, it is about 75.8 years in Tehran province, accounting for a difference of 12.6 % among women [15].
  **Non-communicable diseases:** Women shared 31.2 % of all burden of disease (DALY_S_) of non-communicable diseases (NCDs) in 2003 compared with 32.7 % in 2011, an increase of about 4.8 % [16]. The most prevalent diseases among Iranian females were cardiac ischemia, depressive disorders, and osteoarthritis (Fig. 2).The share of pregnancy and diseases related to childbirth in women was 7 % in 2003, which decreased slightly (0.4 %) to 6.6 % in 2011 [17]. However, the principal cause of mortality in women was cardiac ischemia which caused 24.3 % and 24.69 % of deaths in 2003 and 2011, respectively [16]. The respective values for obesity and overweight with 9.33 % DALY and disability with 11.87 YLD pose the highest associated risk factors for such diseases among Iranian women, followed by dietary risk with 9.19 % DALY combined with 1.69 % disability, high blood pressure with 7.72 % DALY, and 83 % disability [17]. A comparison of differences in risk factors associated with such NCDs between the two genders showed that 20.39 % of men and 1.02 % of women aged 15 to 64 years were smokers. The mean body mass indexes (BMI) were 25.66 in women and 24.22 in men. High blood pressure (systolic blood pressure ≥ 140 and/or diastolic blood pressure ≥ 90 mmHg) presented in 16.12 % of women and 16.07 % of men. Moreover, 49.91 % of women and 28.28 % of men had low physical activity defined as <600 MET-minutes/week [18].

**Fig. 3:**
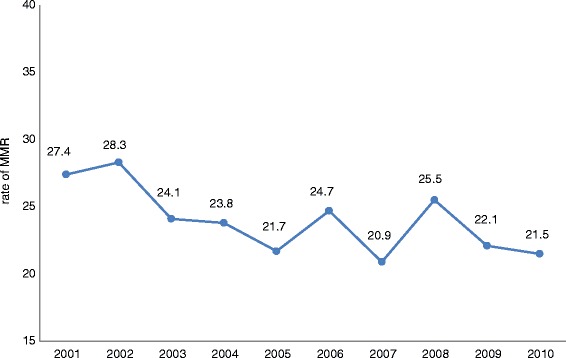
The rate of MMR in (Iran Islamic Republic of) during 2001 to 2010
2. Mental health indicators
  **Psychiatry and depressive disorders:** Studies have shown that mental health indicators in Iran show that men are in better condition than women. Psychological disorders, particularly depressive disorders among women’s burden of disease, ranked second in 2003 and first in 2011. The most prevalent diseases among women were anxiety (19.4 %) and mood disorders (17.3 %) [32]. One noteworthy aspect in the area of mental health is the increasing trend of suicide and suicidal attempts in Iran that varies in relation to gender [33]. Although the rate of committed suicide is greater in men than in women, there is a higher frequency of suicide attempts among Iranian women, a situation showing an increasing trend in recent years [34]. Violence and family disputes are among the most important reasons for attempted suicides, especially among married women [35]. A point worthy of note is the in homogeneity in women’s suicide rates at provincial levels; the rate of failed and successful suicide attempts in women in some provinces, especially western provinces, has become a matter of grave concern. The highest rate of suicide is reported among women in Ilam province where it is 18.7 times the number of suicides among Iranian women and 14.6 times the total number of suicide attempts across Iran [36]. Moreover, 27 % of all suicides in Iran were caused by self-immolation; 71 % of such cases involved women [37].
3. Social health indicators
  **Gender Gap Index:** According to the annual reports of the United Nations, the gender gap index (GGI) has been underlined as an important determinant of human development and an indicator that more explicitly calls attention to women’s problems. This index considers gender gap from four aspects: economic participation and opportunity, educational attainment, health and survival, and political empowerment [38]. In regard to this criterion (shown in Table 1), the latest United Nation’s report in 2013 ranked Iran 130th among 136 countries in GGI (score = 0.584). Iran’s GGI had not followed a consistent trend over the given years, changing from 0.622 in 2000 to 0.584 in 2013 and having 0.0039 as the score for this criterion. On the other hand, 62.1 % of adult women accessed a secondary education or higher compared to their male counterparts [39]. In regard to participation in the job market, 16.4 % and 72.5 % of opportunities are assigned to females and males, respectively [38].

**Table 1:** Iran ranks and scores during 2000-2013 based on Gender Gap Index

	Overall		Economic participation		Educational attainment		Health and survival		Political Empowerment	
	Rank	Score	Rank	Score	Rank	Score	Rank	Score	Rank	Score
Gender Gap Index 2013 (out of 136 countries)	130	0.584	130	0.365	98	0.965	87	0.971	129	0.035
Gender Gap Index 2012 (out of 135 countries)	127	0.593	130	0.412	101	0.953	87	0.971	126	0.035
Gender Gap Index 2011 (out of 135 countries)	125	0.589	125	0.444	105	0.925	85	0.971	130	0.017
Gender Gap Index 2010 (out of 134 countries)	123	0.593	125	0.426	96	0.959	83	0.971	129	0.017
Gender Gap Index 2009 (out of 134 countries)	128	0.584	131	0.377	96	0.964	63	0.978	132	0.017
Gender Gap Index 2008 (out of 130 countries)	116	0.602	118	0.449	92	0.965	60	0.978	128	0.017
Gender Gap Index 2007 (out of 128 countries)	118	0.590	123	0.395	90	0.958	58	0.978	122	0.031
Gender Gap Index 2006 (out of 115 countries)	108	0.580	113	0.359	80	0.954	52	0.978	109	0.031
  According to the 2013 census, the unemployment rate of Iranians 10 years old or older was 10.5 %, and this rate was doubled in females compared to males (18.95 % vs. 9.1 %). The highest rate of unemployment in young people was found in university graduates (37 %) involving 29 % and 48 % of men and women, respectively. Unemployment rates differed in various provinces. The rate of economic cooperation by women in Iran has shown an increasing trend relative to men in recent years. This was shown by an 8.15 % economic cooperation rate of women in 1990 compared to 16.4 % in 2011 [40].
  Basic education is considered an important indicator of empowerment [41]. Iran has witnessed a very rapid growth in literacy during recent years, shown by 28 % to 96 % literacy rates among men and 10 % to 97 % in young women. Women outnumbered men in entering colleges and Iranian universities; this has shown a growth rate of 6.1 % during the period 2006 to 2011. In this context, there are in homogeneities at provincial levels. For instance, the literacy rate of young women is the highest (near 100 %) in Semnan, a middle socio-economic province, compared to Sistan Baluchistan province which has 68.85 % literacy [42]. Another important point was the differences existing between literacy rates in young women living in urban and rural areas, where 5 % of such difference (98 and 93 %, respectively) is still noticeable [43].
  Another gender-related problem in Iran is female heads of household which constitute at least 5.2 million or the equivalent of 12 % of Iranian households. This rate has shown an increasing rate of 72 % over the last 15 years [44]. Economically speaking, female heads of household are more vulnerable than their male counterparts; 43 % of women in this category belong to the two lowest income deciles, whereas men as heads of household constitute only 16 % of this group. The highest rate of breadwinner women (64 %) lived in Sistan-Baluchistan province. Moreover, 9.7 % of female heads of household suffer from disability and psycho-somatic disorders, and 30 % are unable to work because of problems associated with their poor health status [45]. With respect to gender-related poverty, destitution is more pronounced among female household heads than male breadwinners (0.51 % vs. 0.41 %) [46].
  **Women and social violence:** Despite the recently increasing number of women detained for committing various offenses, only 4.48 % of prisoners with criminal offenses constitute women. As for cooperation between the two genders in committing violent crimes (murder), the ratio of women to men is 1:9 [36]. Generally, statistical analyses have shown a wide spectrum of violence toward women in families, ranging from 17.5 % of pregnant women in Khorasan Razavi to 93.6 % of women in Mazandaran province [47]. The results of inter-provincial studies show a generally high prevalence (2.34 DALY) of violence against women in Iranian families, but the prevalence of violence against women was lowest in Tehran province. Studies have also shown that increasing violence is directly related to rising age in women [48]. However, there is an inverse relationship between education levels, especially a university education, and the prevalence of violence. In addition, there is remarkably less violence against working women than housewives [47].
  Studies have shown that the rate of female genital mutilation (FGM) in some ethnicities of Iran is more than 60 %. Pashaei et al. showed that keeping with tradition, cleanliness, religious recommendations, and control of sexual desire were the main reasons for performing FGM. One noticeable point is that less than 3 % of respondents named religion as the reason for continuing FGM to the next generation, a tradition which is inversely related to the level of education [49].

## Discussion

Despite the promising trend of women’s health in Iran over the past three decades, there is still a difference between women and men with respect to mental, physical, and social health. According to the World Development Report in 2012, women in developing nations, unlike those in developed countries, experience greater mortality rates than men [50]. Gender is a critical determinant for health status in many societies and is rooted in social, cultural, political, and health system-related factors [51–53]. Similar to Iranian females, the global burden of diseases also shows that women aged 15 to 65 years lose more healthy life to disability than men. In spite of this fact, disability has been almost neglected as a central policy priority during the era of the Millennium Development Goals (MDG5) [54].

The nearly 75 % decrease in Iran’s maternal mortality (from 83 to 23 %) from 1990 to 2013 is a remarkable achievement in women’s health which correlates with MDG5 goals and is much higher than the global trend (22 %) [55, 56]. There is a correlation between the Human Development Index (HDI) and the socio-economic situation of Iranian females. In Sistan -Baluchestan with an HDI of 0.58, maternal mortality is 53.67 %, a rate much higher than that of Tehran province which has HDI 0.78 and maternal mortality of 11.98 [28, 29]. According to a report by Iran’s Ministry of Health, more than 50 % of this mortality is still preventable. Hemorrhage, eclampsia, and infection constitute about 50 % of the causes of maternal mortality in Iran. The quality of hospital care, on-time referral of high risk pregnant women, and high rate of caesarian section are considered to be the most important pitfalls of Iran’s health system [28].

Mental disorders and domestic violence are other high priority problems among Iranian women. According to a systematic review, social determinants of health have a remarkable relationship with violence not only in Iran, but also on a global scale [57, 58]. According to the 2005 WHO report, the highest rates of violence against women occurred in Bangladesh, Ethiopia, Peru, Tanzania, and to a lesser extent in Japan [59]. According to the last study of burden of disease, non-communicable diseases (NCDs) are a dominant cause of morbidity and disability among Iranian women; the statistics were comparable to many middle income and developed countries [16]. Several factors could account for this phenomenon, of which the two most important are urbanization and change in lifestyle. The ratio of urbanization in Iran has changed from 0.43 to 2.3 between 1980 and 2012. In addition, fast-paced and stressful living conditions [60] have accelerated the nutrition transition that has occurred in Iran during the two past decades [61]. Along with cultural barriers and a shortage of facilities for women’s physical activity [62], these can be significant contributing factors for the increasing trend of NCDs among Iranian women. A national survey in 2010 showed that physical inactivity for women was very common (46.3 %) and ranged from 23 to 65 %, depending on the residential area [63]. However, cigarette smoking as a risk factor for non-communicable diseases is much less in Iranian females than in Iranian males and close to that of other Persian Gulf countries. It has had a decreasing trend during the past decade, especially among school-aged girls [64, 65].

As results of the current study show, Iran is ranked low in the gender gap index. This is in contrast with the fact that 3.1 % of the seats in parliament are assigned to women, although there has been a decreasing trend in this indicator from 0.035 to 0.028 from 2000-2012 [39]. On the other hand, women participate actively in many health-related, non-governmental organizations. Around 100,000 women work voluntarily with health systems to expand healthcare coverage for households and to elevate the health education of the people, especially in deprived areas [66].

According to the reports of international organizations, Iranian women’s health indicators on average are one of the best in the Eastern Mediterranean region [15]. However, this does not mean that the amount of social participation of women in Iran is acceptable. Considering the gender gap index, Iranian females rank low in political and economic participation, although in access to education and health as well as LE they are ranked better [50]. Based on the gender-related development index, Iran ranked 10th among 19 Islamic countries [38]. Although higher education is most important to elevating the position of women in Iran’s society, it can cause scholarly accumulation if not properly applied, resulting in the insufficient participation of women in political and economic spheres [67]. The rate of criminal offenses committed by women has increased proportionately to the rising socio-economic crises and population growth. There has been a steady increase in the number of female offenders compared to men over the past decades, mostly because of drug-related offenses and promiscuity.

## Conclusions

In summary, the challenges to women’s health in Iran are rooted in two main areas. The first comprise the social determinants of health which entitle women to equal social positions with men regardless of socio-cultural classification. Women’s social values and rights vary in different cultures and locations. For instance, in very traditional societies like some ethnicities in Iran, the social rights of men and women are not equal [68, 69]. Moreover, deprivation that contributes to ill health is common in both developed and developing countries. An evident example is the situation of African Americans in the United States who have lower health indicators than whites because of their socio-economic conditions [70]. The second area refers to the health system and its response to women’s needs. Comprehensiveness of a health system is a very important factor for an effective response to the needs of the target population. In this regard, there are two main defects in Iran’s health system. Although mental disorders, breast cancer, and violence are placed at the top of the burden of diseases’ list among Iranian women [16, 71], these priorities have not been seen in the current basic service packages at different levels of Iran’s health system. In addition, the imperfect referral system [72] along with fragmentation between different parts of the health system results in a lack of continuity of care, which, in turn, provokes incomplete results for healthcare [69].

Regarding the issues discussed above, which are comparable to many countries, and in order to address the gender chasm, the first strategy could be legislation and the empowerment of women by providing public policy advocacy to protect effectively females against any shape of discrimination and violence, building up the women’s Non-Governmental Organization for getting their involvement in female related socio- economic issues, and promoting public awareness about women’s right. [71]. The other strategy is Universal Health Coverage which is the most effective remedy for the deficiencies in Iran’s current health system, with an emphasis on providing essential and quality services. This reduces the effect of social determinants of health, which demands specific attention to bolster and improve the primary health care.
